# Network-Based Analysis of Software Change Propagation

**DOI:** 10.1155/2014/237243

**Published:** 2014-03-26

**Authors:** Rongcun Wang, Rubing Huang, Binbin Qu

**Affiliations:** ^1^School of Computer Science and Technology, Huazhong University of Science and Technology, Wuhan 430063, China; ^2^School of Computer Science and Telecommunication Engineering, Jiangsu University, Zhenjiang, Jiangsu 212013, China

## Abstract

The object-oriented software systems frequently evolve to meet new change requirements. Understanding the characteristics of changes aids testers and system designers to improve the quality of softwares. Identifying important modules becomes a key issue in the process of evolution. In this context, a novel network-based approach is proposed to comprehensively investigate change distributions and the correlation between centrality measures and the scope of change propagation. First, software dependency networks are constructed at class level. And then, the number of times of cochanges among classes is minded from software repositories. According to the dependency relationships and the number of times of cochanges among classes, the scope of change propagation is calculated. Using Spearman rank correlation analyzes the correlation between centrality measures and the scope of change propagation. Three case studies on java open source software projects Findbugs, Hibernate, and Spring are conducted to research the characteristics of change propagation. Experimental results show that (i) change distribution is very uneven; (ii) PageRank, Degree, and CIRank are significantly correlated to the scope of change propagation. Particularly, CIRank shows higher correlation coefficient, which suggests it can be a more useful indicator for measuring the scope of change propagation of classes in object-oriented software system.

## 1. Introduction

During the whole life cycle of object-oriented software systems, frequent change of software artifacts is an eternal theme, especially in maintenance and evolution phases. Developers must modify software entities such as functions, variables, or interfaces frequently to meet new requirements, for example, upgrading the software system or fixing newly found bugs [1]. Generally speaking, these modifications introduce new changes to a system. Moreover, these new changes may influence other existing modules, which may produce undesirable consequences, for example, injecting a new defect, breaking existing functionality, or decreasing the performance of the application [2]. This phenomenon is called “change propagation” or “ripple effects” [3, 4].

Change impact analysis is an active topic in software engineering community. Many research efforts [1, 2, 5–8] have been made to predict the possibly affected scope at different levels of software, for example, source code, requirements, and architectural models. However, few studies attempted to identify the key classes in the process of software evolution or characterize change distributions in complex software systems. Particularly, with the increasing scale of object-oriented software systems, it is much more difficult for developers and testers to identify the scope of change impact. In view of the side effects generated by changes, it is essential to intensively investigate the characteristics of change propagation and distributions. The results we studied would enable the project managers and developers to estimate the efforts of software maintenance more accurately and distribute the workload in a more reasonable manner. In particular, it aids testers and system designers to identify those modules whose changes have important influence on the reliability of the software system in regression testing.

In the object-oriented software system written in Java, the whole system can be modularized into classes. The classes and structural code dependency relationships among classes are modeled into software dependency network. Intuitively speaking, the changes of different classes produce the different effects on the whole system. Ranking classes with respect to the scope of their change propagation become a complicate issue. Unfortunately, there is still a research gap on how to rank classes based on the importance of change propagation. Fortunately, in coauthorship networks, the popularity and prestige of journals and authors are ranked according to the value of network centrality [9]. The application of centrality measures to coauthorship networks provides a new explication for our study. Can these centrality measures be applied for software dependency networks to rank classes based on the scope of change propagation of classes? In other words, are network centrality measures collected with the scope of change propagation?

In this context, this paper proposes a network-based approach to study change propagation. Log information of historical changes of classes extracted from CVS is analyzed to calculate the number of times of cochanges among classes. Structural code dependency relationships at class level are combined with the relations of cochange among classes to calculate the scope of propagation in the software system. In this paper, we conducted three case studies on more than twenty consecutive releases for three Java open source projects Findbugs, Hibernate, and Spring Framework, respectively, to validate the relations between the centrality measures and the scope of change propagation. Information on structural code dependency relationships and historical changes of 4000 classes was collected and analyzed so as to answer the following two key questions.

**Question 1**

What characteristics do change distributions have in software evolution phase?

**Question 2**

Can network centrality measures be applied to rank classes based on the scope of change propagation? Which centrality measure is more correlated with the scope of change propagation of classes?

The remainder of this paper is organized as follows.  Section 1 summarizes related works to change impact analysis and the applications of centrality measures. The related concepts of network theories are discussed in Section 3. A network-based approach is proposed with a new centrality measure CIRank for measuring the scope of change propagation in Section 4. The experimental design and the case studies are presented in Section 5. In Section 6, experimental results are analyzed and the threats to validity of the study are discussed in Section 7.  Section 8 describes the conclusions and future work.

## 2. Related Works

In this section, we discuss related works to our study including change impact analysis and centrality measure.

### 2.1. Change Impact Analysis

Zimmermann et al. [5] applied association rules to mine evolutionary coupling between fine-grained program entities such as functions or variables. They also implemented a tool named ROSE that can correctly predict further locations affected by an initial change. Similarly, Ying et al. [10] also applied data mining techniques to determine change patterns between classes.

Structure-based techniques were widely applied to change impact analysis, mainly including program dependency graphs [7], call graphs [11, 12], and message dependency graphs [13–15]. Popescu [14] showed a Helios approach to determine message dependency graphs. Moreover, the approach was also applied to event-based applications for change impact analysis. Badri et al. [12] proposed a model based on method-level control call graphs to predict change impact. In contrast with the traditional call graphs, the approach provided more accurate prediction results as it considered the control related to the calls between components. German et al. [16] assessed the impact of historical code changes on a particular code segment by visualizing the impact into change impact graphs, which were then pruned to rapidly pinpoint the source of a bug.

Different slicing techniques were widely applied to change impact analysis, such as static slicing [2, 17], dynamic slicing [18], thin slicing [19], process slicing [20], and probabilistic slicing [21]. Similarly, Bayesian Belief Network was applied by Tang et al. [8] to quantify the likelihood of change impact based on the dependency information between architecture decisions and elements. Wong and Cai [22] proposed a stochastic dependency framework based on the Markov chain model to compute the probability that two components are logically dependent. The probability value was used to predict whether a component would be affected by a change.

More recently, information retrieval methods were applied to change impact analysis [23–25]. Hassaine et al. proposed a seismology-inspired approach to study change propagation, that is, how far a change propagation will proceed from a given class to the others [26]. Kapodjedo et al. proposed several design evolution metrics to predict defects in object-oriented systems. These metrics were applied to recommend a ranked list of classes potentially containing defects [27].

### 2.2. Centrality Measure

Centrality measures originated from the research community on social network as Freemann [28] developed a set of measures of centrality based on betweenness. These centrality measures have since been further developed into degree centrality, closeness centrality, betweenness centrality, and eigenvector centrality. More recently, centrality measures have been applied in other fields such as technological networks [29] and coauthorship networks [9]. Additionally, Hackman [30] discussed the relationship between centrality and power in the allocation of resources in universities. Bonacich [31] used power and centrality to measure a family. As for coauthorship networks, centrality measures were applied to indicate popularity rank and prestige rank of journals and authors [9].

Zimmermann and Nagappan [29] used network analysis on code dependency graphs to identify central program units that are more likely to face defects. Pinzger et al. [32] also empirically investigated centrality measures that were significant to predict the probability and number of postrelease failures in developer contribution network. Similarly, centrality measures are also applied to our study to rank classes that have important influence on the reliability of system.

## 3. Network Theories

Centrality measures are used to reflect the importance or prominence of nodes in the network. The more central a node is in a network, the more significant it is to spread information. In general, there are two types of centrality measures: the micro centrality measures concerned with the ranking of individual nodes or edges based on the topology structure of network; the macro centrality measures describing the general network features.

### 3.1. Micro Centrality Measurements

The micro centrality measures including degree of nodes, closeness, and betweenness, PageRank are used to validate the correlation between them and the scope of change propagation.

#### 3.1.1. Degree Centrality

The degree of a node, denoted by deg⁡(*v*), measures the number of edges that the node has to other nodes where the edges between nodes are either directed or undirected. For directed network, the edges are further divided into indegree edges and outdegree edges. The indeg(*v*), the outdeg(*v*), and the deg⁡(*v*) represent the number of ingoing edges, the number of outdegree edges, and the sum of indeg(*v*) and outdeg(*v*), respectively.

Generally speaking, the nodes with higher degree or more connections have more influence or are likely to be more central than the nodes with lower degree. A node with higher outdegree is more strongly affected by the changes of other nodes. Therefore, the probability of a node to be affected by a change increases with its outdegree.

#### 3.1.2. Closeness Centrality

In social network analysis and other network studies, closeness centrality is widely used to measure the mean distance from a node to all the other nodes. The closeness centrality of node *n*_*i*_ is defined as

(1) $$\begin{array}{c} C_{c}\left(n_{i}\right)=\frac{1}{\sum_{j=1,j\neq i}^{N}d\left(n_{i},n_{j}\right)}, \end{array}$$

where *d*(*n*_*i*_, *n*_*j*_) is the geodesic distances through a network between node *n*_*i*_ and *n*_*j*_ node and *N* represents the number of nodes in the network.

The above definition of closeness centrality can be only applied to strongly connected graph. If there is no path from node *n*_*i*_ to node *n*_*j*_, *d*(*n*_*i*_, *n*_*j*_) will be *∞*. In this case, (1) would not be applied. Therefor, it is necessary to redefine closeness as

(2) $$\begin{array}{c} C_{c}\left(n_{i}\right)=\overset{N}{\underset{j=1,j\neq i}{\sum }}\frac{1}{d\left(n_{i},n_{j}\right)}. \end{array}$$

When node *n*_*i*_ cannot reach *n*_*j*_, the corresponding term in the sum is simple zero and can be eliminated. In the study, software dependency network is not strongly connected graph, so (2) is used.

#### 3.1.3. Betweenness Centrality

Betweenness proposed by Freemann is another node property related to centrality. Communication between nonadjacent nodes might depend on the other nodes, which pass information and control the communication between nonadjacent nodes. Betweenness measures the probability of a node lying on geodesic paths between all other nodes. The betweenness centrality of node *n*_*i*_ is quantitatively defined as

(3) $$\begin{array}{c} C_{b}\left(n_{i}\right)=\underset{\begin{array}{c} j\neq k\neq i \end{array}}{\sum }\frac{g_{jk}\left(n_{i}\right)}{g_{jk}}, \end{array}$$

where *g*_*jk*_(*n*_*i*_) represents the number of geodesic paths between the node *n*_*j*_ and node *n*_*k*_ passing through node *n*_*i*_, and *g*_*jk*_ represents the number of geodesic paths between the node *n*_*j*_ and *n*_*k*_ node.

Nodes with higher betweenness may play a more important broker role on communications between nodes within a network. The removal of the nodes with higher betweenness will also most severely impair communications between others nodes.

#### 3.1.4. PageRank

PageRank is a link analysis algorithm based on web graph, which was developed by Brin and Page [33]. The original PageRank algorithm is applied in web ranking technology to calculate the rank value that indicates the importance of a particular page. A page that is linked by many pages with high PageRank receives a high rank itself. In the case of two pages with the same outdegree, the page with high PageRank contributes more centrality than the other page with low PageRank. The PageRank of node *n*_*i*_ is defined as in the following:

(4) $$\begin{array}{c} \text{PR}\left(n_{i}\right)=\frac{1-d}{N}+d\underset{n_{j}\in M(n_{i})}{\sum }\frac{\text{PR}\left(n_{j}\right)}{\text{outdeg}\left(n_{j}\right)}, \end{array}$$

where *M*(*n*_*i*_) is the set of pages that point to page *n*_*i*_, outdeg(*n*_*j*_) represents the number of outgoing links of page *n*_*j*_, *N* is the total number of pages, and *d* is the damping factor and is usually set to 0.85. Additionally, there are some other centrality measures including eigenvector centrality [31] and the HITS ranking algorithms [34].

## 4. Methodology

To study change propagation, change log information of class files was collected from the source code control repositories and saved to mysql database. In the database, each change of a class file is logged as a record. The dependency relationships between classes were extracted to construct the software dependency networks. In this section, the change log information and structural dependency relationships between classes were combined to study the two questions raised in Section 1.

### 4.1. Cochanges Data Collection

In large software, it is very important to track and understand the characteristics of those changes. In this study, the focus is on change propagation between classes. Changes such as revising copyright information that do not cause ripple effects are beyond the scope of this study. In practice, version control system, for example, CVS and SVN plays a fundamental role in software maintenance and evolution. The repository of CVS stores the historical releases of a project with RCS (revision control system) file format. Once a file is changed, it is stored as a new release in the repository. Therefore, the repository provides change log information of each file in detail including file name and description information. The description information mainly contains revision number, date, author, lines of added codes, lines of removed codes, explanations of change, and so forth.

To collect cochanges classes, we define a quadruple Φ = {CN, AN, CT, *M*} to represent the change set of all classes, where CN represents the set of class name, AN represents the set of author name, that is, the developers participating in projects and doing check-in, CT represents the set of committing time, and *M* represents the set of log messages. In ∀*ϕ*_*i*_ ∈ Φ, *ϕ*_*i*_ represents the class *ϕ*_*i*_(cn) that is committed to sever by the author *ϕ*_*i*_(an) at the time *ϕ*_*i*_(ct), and the description information of *ϕ*_*i*_(cn) is *ϕ*_*i*_(*m*).

If two classes are frequently changed together during development and maintenance, an implicit dependency is assumed. Cochanges of classes are defined as follows.

**Definition 1 (cochanges of classes, CCOC)**

We say that *ϕ*_*i*_(cn) and *ϕ*_*j*_(cn) are evolutionary coupling if and only if ∀*ϕ*_*i*_, *ϕ*_*j*_  (*i* < *j*)  (1)  *ϕ*_*i*_(cn) = *ϕ*_*j*_(cn);  (2)  *ϕ*_*i*_(an) = *ϕ*_*j*_(an);  (3)  *ϕ*_*i*_(*m*) = *ϕ*_*j*_(*m*); and (4)  |*ϕ*_*i*_(ct) − *ϕ*_*j*_(ct) | ≤ Δ*t*. When the all above conditions hold, we have *ϕ*_*i*_(cn) CCOC *ϕ*_*j*_(cn).

Due to that the length of time is not always identical between two-sequent-change committing, a time window Δ*t* is equal to the mean value between two-sequent-change committing.

### 4.2. Software Dependency Network

As a part of object-oriented programming language, Java is very popular in open source community. Consequently, plenty of examples that contain the complete source code evolution can be easily obtained online in software repositories and web development platform, such as SOURCEFORGE (http://www.sourceforge.net/), MVN Repository (http://mvnrepository.com/), and GitHub (https://github.com/).

We mainly focus our efforts on open-source software developed in Java. A method calls another method or a class aggregates the objects of another class—all these come into a direct dependency relationship between two classes. Structural dependency relationships between classes are discussed. The relationships mainly include the following:

1. a class extends another class or implements the interfaces;
2. a class calls the methods provided by other classes;
3. a class references the members of other classes;
4. a class uses other classes as its variables or members.

The object-oriented software is modeled as software dependency networks extracted by Dependency Finder (http://depfind.sourceforge.net/), an open source analysis tool.

**Definition 2 (software dependency network, SDN)**

A directed graph *G* = (*N*, *E*) is defined to represent the software dependency network, where *N* is the set of nodes and *E* is the set of directed edges.

**Figure 1:**
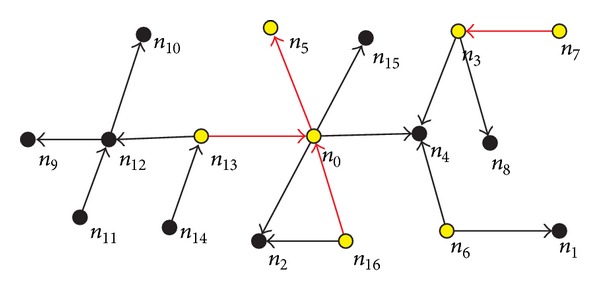
Software dependency network.

In Figure 1, classes are represented as nodes and code dependency relationships between classes as directed edges between nodes. There exists a directed edge *e*  (*e* ∈ *E*) from node *n*_0_ to node *n*_5_; that is, node *n*_0_ depends on node *n*_5_. For the rest of this paper, the terms node and class are interchangeable. This study only concerns how a single change of one class influences other classes. In other word, the self-effects of classes are not considered, so the graph does not contain self-loops.

### 4.3. Combining Cochange with Change Dependency

The method of mining has been used to understand the class evolutionary changes and predict source code change [5, 10], but the dependency relationship between evolutionary coupling classes was ignored. Many researchers believe that the dependencies between modules are particularly relevant as changes propagate along exactly these structure dependencies [1, 35, 36]. Based on the above analysis, we combine cochanges information with dependency relationship between classes to study the change propagation in software dependency network.

#### 4.3.1. Identification of Focus Class

**Definition 3 (focus class)**

Let *θ*  (*θ* ⊂ Φ) represent the set of evolutionary coupling within the same time window Δ*t*. A class is called to the focus class in software dependency network if and only if  ∀*ϕ*_*i*_, *ϕ*_*j*_ ∈ *θ*  (*i* ≠ *j*)  (1)  ∃*R*(*ϕ*_*j*_(cn), *ϕ*_*i*_(cn)) and *ϕ*_*i*_(cn)CCON*ϕ*_*j*_(cn);  (2)  ∀*ϕ*_*k*_ ∈ *θ*  (*i* ≠ *j*), ¬(∃*R*(*ϕ*_*i*_(cn), *ϕ*_*k*_(cn))) where *ϕ*_*j*_(ct) − *ϕ*_*i*_(ct) ≤ Δ*t* and *ϕ*_*j*_(ct) ≥ *ϕ*_*i*_(ct). When all the above conditions hold, *ϕ*_*i*_(cn) is considered to be a focus class.

In Figure 1, all yellow nodes stand for changed classes within the same time window Δ*t*, while black nodes stand for unchanged classes. The arcs between nodes represent dependency relationships of nodes, for example, the arc from node *n*_0_ to node *n*_2_ shows that node *n*_0_ depends on node *n*_2_. Within the same time window Δ*t*, nodes *n*_0_, *n*_3_, *n*_5_, *n*_6_, *n*_7_, *n*_16_, and *n*_13_ were changed. According to the Definitions 3 and 4 change dependencies relationships are shaped, that is, (*n*_5_-*n*_0_-*n*_16_), (*n*_5_-*n*_0_-*n*_13_), (*n*_7_-*n*_3_), and (*n*_6_). The change of node *n*_7_ is affected by the change of node *n*_3_. Node *n*_3_ is considered as a focus class. In the same way, node *n*_5_ is also considered as a focus class.

#### 4.3.2. The Scope of Change Propagation

In software dependency network, when one node is changed, we only consider the nearest neighbors that can be affected by the node. In this sense, it is relatively easy to locate the changed nodes. However, as shown in Figure 1, changes do propagate further than their direct neighbors. In this case, the effect of changes may propagate further than their immediate neighbors; that is, if a node is changed, all those nodes that point to it directly or indirectly may be also affected. In contrast to the first case, it may be more difficult to locate those affected nodes, especially for those far from the root nodes. Therefore, the study synthetically takes into account the depth and breadth of change propagation for measuring the scope of change propagation of a node. Let *ℓ*_*ij*_ denote the shortest path length from node *n*_*j*_ to node *n*_*i*_. The length of *ℓ*_*ij*_ is equal to the number of edges from node *n*_*j*_ to node *n*_*i*_. Denote PC′(*n*_*i*_) as the scope of change propagation of node *n*_*i*_ in a time change propagation. PC′(*n*_*i*_) is defined as follows:

(5) $$\begin{array}{c} {\text{PC}}^{\prime }\left(n_{i}\right)=\overset{N}{\underset{j=1,j\neq i}{\sum }}\ell_{ij}, \end{array}$$

where *N* denotes the number of nodes that can reach node *n*_*i*_ in software change propagation network. Node *n*_*i*_ as a focus class may appear *κ* times in *N* times change. For a focus class, its propagation capability may be very strong. At times, the propagation capability is weak. Consequently, the study takes the average value of PC′(*n*_*i*_) to more objectively evaluate the scope of change propagation of node *n*_*i*_:

(6) $$\begin{array}{c} \text{PC}\left(n_{i}\right)=\frac{\sum_{m=1}^{\kappa }{\text{PC}}^{\prime }{\left(n_{i}\right)}_{m}}{\kappa }. \end{array}$$

#### 4.3.3. Ranking Change Propagation of Class

Change propagation in software dependency network is very similar to navigation between web pages. In software dependency network, change propagates along the reverse direction of the dependency between nodes. However, PageRank propagates centrality along the direction of the edges. Additionally, for a page, PageRank treats PageRank score of every incoming with equal weight. In software dependency network, the frequency of cochanges between different node pairs is different. Therefore, for a node, the study differentiates the change propagation from its outgoing according to different weights of edges determined by the frequencies of cochanges between the nodes.

**Definition 4 (weight factor *ω*_*ij*_)**

Node *n*_*i*_ depends on node *n*_*j*_. Let *D*(*n*_*j*_) denote the set of nodes that depends on node *n*_*j*_. CCN(*n*_*i*_, *n*_*j*_)  (CCN(*n*_*i*_, *n*_*j*_) ≥ 1) is denoted by the cochanges number of node *n*_*j*_ and node *n*_*i*_. Weight factor is defined as in the following:

(7) $$\begin{array}{c} \omega_{ij}=\frac{\text{CCN}\left(n_{i},n_{j}\right)}{\sum_{k\in D(n_{j})}^{}\text{CCN}\left(n_{k},n_{j}\right)}, \end{array}$$

where the value of *ω*_*ij*_ lies in the range 0 < *ω*_*ij*_ ≤ 1. If there is no cochange between node *n*_*i*_ and *n*_*j*_, CCN(*n*_*i*_, *n*_*j*_) is equal to 1. Based on the above analysis, we introduce a measure called change impact rank (CIRank), which is based on the PageRank algorithm. Similar to PageRank, the algorithm of CIRank is convergent, when the ranking value of every node in the software dependency networks has no further change. The probability of a node being infected by the changes of its adjacent nodes is related to not only the number of its outgoing edges, but also the probability of its adjacent nodes being infected. The change impact rank of node is defined as follows:

(8) $$\begin{array}{c} \text{CIR}\left(n_{i}\right)=\frac{1-d}{N}+d\underset{n_{j}\in M(n_{i})}{\sum }\frac{\text{CIR}\left(n_{j}\right)}{\text{indeg}\left(n_{j}\right)}\omega_{ij}, \end{array}$$

where *M*(*n*_*i*_) is the set of nodes whose changes would impact node *n*_*i*_ (|*M*(*n*_*i*_)| = outdeg(*n*_*i*_)); indeg(*n*_*j*_) represents the number of ingoing links of node *n*_*j*_; *ω*_*ij*_ is weight factor of the links from node *n*_*i*_ to node *n*_*j*_.

In general, it is more likely for change to propagate along high weight factor edges than low weight factor edges. In Section 6, the superiority of CIRank to PageRank will be shown. Equation (8) considers not only structural code dependency relationships between nodes, but also the frequencies of cochanges between adjacent nodes. We analyzed how well different centrality measures serve as an indicator for the scope of change propagation of focus classes in software dependency network.

## 5. Empirical Studies

One of the objectives of our study is to characterize change propagation in software during software evolution phrase through analyzing large software projects. The study aims at answering the two questions proposed in Section 1.

### 5.1. Subject Projects

We collected more than 20 consecutive releases for three Java-based open source software projects FindBugs, Hibernate, and Spring Framework from online software repositories Source Forge. FindBugs (http://findbugs.sourceforge.net/) is a free tool developed in Java, which is used to look for the bugs in Java code through static analysis. Hibernate (http://sourceforge.net/projects/hibernate/) provides a highly efficient object/relational persistence and query service, which focuses on the mapping from Java classes to database tables and from Java data types to SQL data type. Spring Framework (http://www.springsource.org/) is a light weight and open source framework, which can help development teams focus only on application layer business logic without relying on the special development scenario. Descriptive information about the three selected projects is presented in Table 1.

### 5.2. Experimental Setup

Our experiment mainly includes four steps.

**Step 1 (extracting the set of cochange classes)**

Since the study primarily concerns the changes of class files, other type files (e.g., xml, html, and rtf) are filtered. Additionally, text information of change description is analyzed while those classes not causing change propagation are excluded. All records in Φ are divided into groups by using time window.

**Step 2 (constructing software dependency network)**

Software dependency network is constructed for each release of three subject projects. The network is expressed as a directed graph with GraphML (http://graphml.graphdrawing.org/) file format. The format is chosen because it easily describes the structure properties and can be conveniently extended. Attribute information stored in nodes and edges is shown in Table 2.

**Step 3 (change distributions)**

To characterize the change distributions, we have to calculate the cumulative times of changes of each class in all releases. It is hypothesized that a smaller number of files contain the most of the changes during the evolution phrase of software system. In order to verify our hypothesis, the study employs Alberg diagrams [37] to report our change distributions during software evolution.

**Step 4 (correlation analysis)**

For each release, all focus classes are ranked in descending order of the scope of change propagation. The five centrality measures are calculated in each release, and focus classes are also ranked based on the descending order of their centrality. Using Spearman rank correlation analysis, the coefficients between five centrality measures and the scope of change propagation are calculated to verify which centrality measurements are more related to the scope of change propagation.

## 6. Experimental Results and Analysis

### 6.1. Change Distributions in Software System

The study about change distributions is inspired by Pareto principle (also known as the 80-20 rule), originated in economics, which has been widely applied in computer science and engineering control theory. The study employs Alberg diagrams to observe whether the change distributions can be explained by Pareto principle; that is, 20 percent of class files in software system account for 80 percent of changes during the software evolution. The class files are sorted in decreasing order according to the number of changes only including the changes in the current researched releases. The relationship of class files versus changes is represented in percentage scale. Additionally, if these 20% class files account for the majority of system sizes, the applicability of Pareto principle would be meaningless. Therefore, this study calculates the lines of code of the top 20% of the class files.

#### 6.1.1. Experimental Results

**Figure 2:**
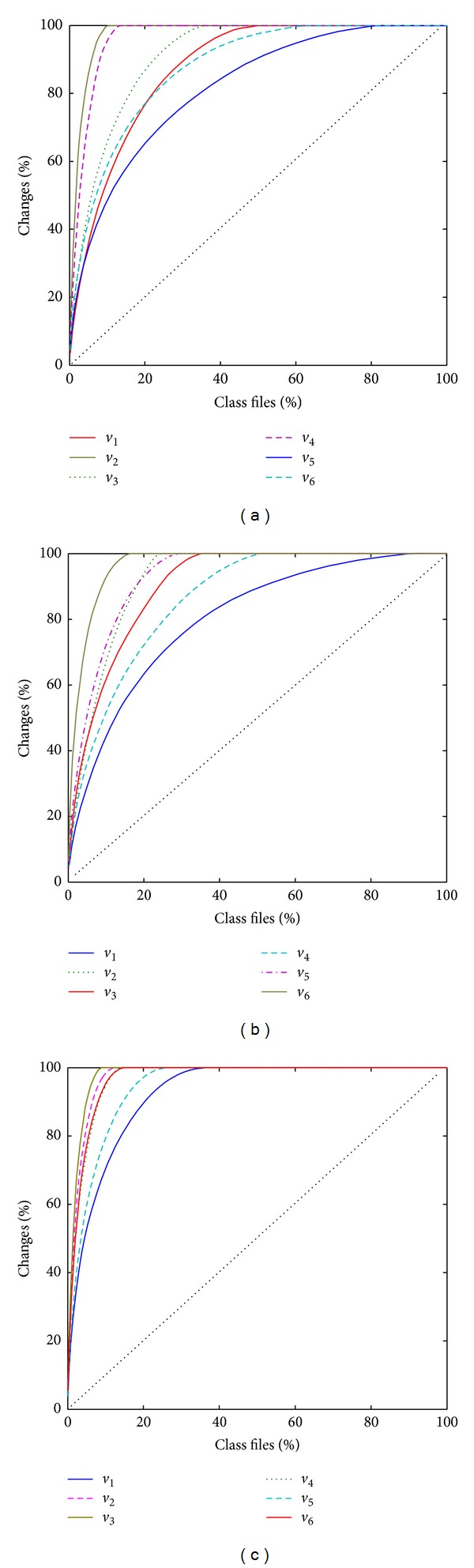
Albert diagram of change distribution.

Figure 2 shows the Alberg diagram of change distribution in the three subject projects. The horizontal axis represents the cumulative percentage of class files, and the vertical axis represents the cumulative percentage of changes. The experiment collected more than twenty releases for three subject projects. As the changes distribution curves for different releases are overlapped in the same Albert diagram, only 6 releases for three subject projects were selected, respectively, in order to clearly show the changes distribution curves. Without loss of generality, for each subject project, the curves with the greatest and smallest deviation from the diagonal line were selected as the upper and lower bounds of the cumulative changes distribution curve, respectively. Four other releases were then selected from the rest of the releases.

#### 6.1.2. Experimental Analysis

As a reference, the dotted line (diagonal line) marks the line of perfect equality. The closer the curve is to the diagonal line, the more uniform the changes distributions are. As this grey green solid line is more strongly bent than other curves, the changes distribution of the grey green solid line is the least uniform. The Alberg diagrams indicate that the most active 10 percent of the class files are responsible for over 50 percent of the changes in all the three projects. In 6 releases of Findbugs, the most active 20% of class files account for 76%, 100%, 86%, 100%, 65%, and 77% of changes, respectively. Hibernate and Spring show the same change distribution with Findbugs. In 6 releases of Findbugs, 20% of change prone class files account for 25%, 31%, 29%, 26%, 30%, and 28% of the code size, respectively. Similarly, in Hibernate and Spring, 20% of change prone class files share no more than 36% of the code size. The results provide strong support for the applicability of Pareto principle to the hypothesis stated in Section 5.

### 6.2. Correlation Analysis

Spearman correlation is used to investigate the correlation between centrality measures and the scope of change propagation. The coefficients which range from −1 to 1 reflect how closely the variables are related. The larger the absolute values of the coefficients are, the more closely the variables are associated.

The releases in this analysis are denoted as *R*_1_, *R*_2_,…, *R*_*n*_. In view of space limitation, only partial correlations are shown in Table 3. The following observations can be made.

1. Betweenness measure shows lower correlation with the scope of change propagation of focus classes. A possible explanation is that those nodes with higher betweenness play the roles of brokers, while they rarely act as focus classes. Additionally, the length of change propagation path (the maximum hops from focus class to the nodes affected by the focus classes) is usually less than 4. In most cases, the length is less than 2. In other words, many changes of the nodes directly propagate to their neighbors without the help of brokers.
2. Closeness centrality has slightly negative correlation coefficient with the degree of change propagation in most cases. The primary reason may be that changes propagate along the reverse direction of dependency, but closeness centrality is calculated along the direction of dependency.
3. Network measures including Degree, PageRank, and CIRank have higher correlation coefficients with the scope of change propagation than closeness and betweenness. Among the first three measures, the most relevant is CIRank, followed by PageRank and Degree. Since Degree centrality only considers the local characteristic of node, it neglects the mediate propagation of changes. Although PageRank centrality considers the global characteristic of nodes, it treats each edge with equal weight.
4. CIRank centrality not only considers the number of outgoing edges, but also differentiates the weights of outgoing edges. The correlation between change propagation and CIRank centrality is the highest observed correlation. This suggests that CIRank can be used to evaluate change impact of classes more effectively.
5. Centrality measures including Degree, PageRank, and CIRank can be applied to evaluate change propagation of classes in the software dependency networks. The application of centrality measures is also related to the networks structure.

### 6.3. Evaluation of Centrality Measures

According to the scope of change propagation, top 5, 10, 20, and 50 focus classes are selected to validate the power of ranking.  Table 4 shows the number of the hitting focus classes. CIRank shows higher hit rate than other centrality measures. The results imply that CIRank can be applied to more accurately rank focus classes according to the scope of change propagation.

## 7. Threats to Validity

Although the experiments were carefully designed, the present study is not free from threats to its validity. These threats to validity can be summarized as follows.

### 7.1. External Validity

The external validity of the study is compromised by the nature and the size of the subject projects, which are all open-source software projects written in Java. As described in Section 5, the three subject projects varied in size and development periods. Although the subject projects studied spanned over 8 years, they may be not necessarily representative for all different projects. We cannot assume that the results are applicable to other software projects as different software evolution might possibly lead to different change propagation.

### 7.2. Internal Validity

The issue of internal validity of the study is related to the length of time window. A too long time window would affect our experimental results, which may magnify the scope of change propagation of focus classes. However, a too narrow time window would impair the effect of the focus classes.

## 8. Conclusions and Future Work

The researched results can be used to provide decision support for project managers on how to assign testing resources and also aid testers to prioritize testing those modules based on centrality measures. To the best of our knowledge, there are few studies investigating the characteristics of change distribution and how to identify those key classes with important influence on the reliability of system.

### 8.1. Conclusions

In this paper, we proposed a novel network-based approach to study change propagation. Three case studies were conducted and made the following observations.

1. A small number of class files contain most of changes. The uneven distribution of changes stimulates unequal distribution of software maintenance efforts.
2. In correlation analysis, closeness centrality shows slightly negative correlations with degree of change propagation, and betweenness centrality shows slightly positive correlations.
3. With regard to Degree, PageRank, and CIRank centrality, they are significantly correlative with the scope of change propagation. Since CIRank centrality considers outdegree of nodes and weight, in generally, it shows stronger correlation than other centrality measures.

### 8.2. Future Work

For future work, our study will be extended to other projects written in non-Java languages, either open-source or proprietary to confirm our results. Different change events might produce different effects on the whole software system. Therefore, change propagation based on change events will be further explored. Frequent subgraph mining technology will be also investigated to discover change propagation patterns in software change propagation network. Last but not least, in regression testing, the idea of ranking classes based on centrality will be confirmed to reduce software maintenance efforts.

## Figures and Tables

**Table 1:** Descriptive information for subject projects.

Program	Start dates	End dates	Last release
FindBugs	2003-08-19	2011-12-21	2.0.1
Hibernate	2002-12-08	2011-12-16	4.1.1
Spring	2004-01-05	2012-02-16	3.2.0

**Table 2:** Descriptive information for GraphML file.

Object	Attribute	Description
Graph	edge default	The type of edge

Node	name	The name of node
	is changed	In current release whether the node is changed

Edge	name	The name of edge
	source	The source node name
	target	The target node name
	ccount	The cochange number in current release
	tccount	The cumulative cochange number in current release and historical release

**Table 3:** Spearman's coefficient for three subject projects.

Projects	Centrality	The scope of change propagation of focus class							
Findbugs (*R*_1_,…, *R*_8_)	Degree	0.3841	0.6790**	0.2355	0.7294**	0.2534	0.6079**	0.7096**	0.5625**
	Closeness	−0.0863	−0.2962**	−0.3017	−0.5201**	−0.4662**	−0.2428**	−0.1710	−0.1912**
	Betweenness	0.2554	0.4567**	0.0982	0.3463	0.2313	0.3312**	0.3832**	0.2826**
	PageRank	0.4713*	0.7421**	0.3949*	0.7902**	0.5705**	0.6254**	0.6491**	0.5903**
	CIRank	0.5268*	0.8215**	0.5623**	0.8134**	0.5736**	0.7189**	0.7523**	0.7082**

Hibernate (*R*_1_,…, *R*_8_)	Degree	0.3125**	0.3571**	0.6275**	0.2283**	0.3457**	0.3592**	0.3234**	0.5126**
	Closeness	0.0811	0.0866	−0.2240**	0.0661	0.1292**	−0.1909**	−0.1217**	0.0189
	Betweenness	0.1350	0.1607*	0.4854**	0.1143	0.1932**	0.2712**	0.2214**	0.2883**
	PageRank	0.3538**	0.4069**	0.6937**	0.2950**	0.4157**	0.4216**	0.3302**	0.5090**
	CIRank	0.4463**	0.4890**	0.7315**	0.3247**	0.4862**	0.5139**	0.4153**	0.5672**

Spring (*R*_1_,…, *R*_8_)	Degree	0.5935**	0.5855**	0.6357**	0.5221**	0.2773**	0.4503**	0.6985**	0.5352**
	Closeness	−0.2635**	−0.2258**	−0.2914**	−0.2934	−0.4422**	−0.1907**	−0.1237	−0.2392*
	Betweenness	0.4048**	0.5085**	0.3015**	0.4769**	0.1567**	0.2876**	0.3964**	0.2486*
	PageRank	0.6898**	0.6838**	**0.6687**	0.6970**	0.6918**	0.6874**	0.7542**	0.6524*
	CIRank	0.7250**	0.7563**	**0.6918**	0.8073**	0.7519**	0.7348**	0.8124**	0.7351**

Note. Correlations larger than 0.05 are printed in boldface. Correlations are significant at the 0.01 level (2-tailed) and are marked by ∗∗. Correlation coefficients are significant at 0.05 level (2-tailed) and are marked by ∗.

**Table 4:** Centrality measures ranking data.

Centrality	Top 5	Top 10	Top 20	Top 50
Degree	2	4	11	30
Closeness	1	2	6	16
Betweenness	2	3	8	19
PageRank	3	6	12	32
CIRank	4	8	15	40
